# Direct fishing and eDNA metabarcoding for biomonitoring during a 3-year survey significantly improves number of fish detected around a South East Asian reservoir

**DOI:** 10.1371/journal.pone.0208592

**Published:** 2018-12-13

**Authors:** Benjamin Gillet, Maud Cottet, Thibault Destanque, Kaoboun Kue, Stéphane Descloux, Vincent Chanudet, Sandrine Hughes

**Affiliations:** 1 Institut de Génomique Fonctionnelle de Lyon, Université de Lyon, Ecole Normale Supérieure de Lyon, Centre National de la Recherche Scientifique, Université Claude Bernard Lyon 1, Unité Mixte de Recherche, Lyon, France; 2 Nam Theun 2 Power Company Limited, Environment & Social Division, Environment Department, Gnommalath Office, Vientiane, Lao PDR; 3 EDF, Hydro Engineering Centre, Environment and Social Department, Le Bourget-du-Lac, France; University of Guelph, CANADA

## Abstract

Biodiversity has to be accurately evaluated to assess more precisely possible dam effects on fish populations, in particular on the most biodiverse rivers such as the Mekong River. To improve tools for fish biodiversity assessment, a methodological survey was performed in the surroundings of a recent hydropower dam in the Mekong basin, the Nam Theun 2 project. Results of two different approaches, experimental surface gillnets capture and environmental DNA metabarcoding assays based on 12S ribosomal RNA and cytochrome *b*, were compared during 3 years (2014–2016). Pitfalls and benefits were identified for each method but the combined use of both approaches indisputably allows describing more accurately fish diversity around the reservoir. Importantly, striking convergent results were observed for biodiversity reports. 75% of the fish species caught by gillnets (62/82) were shown by the metabarcoding study performed on DNA extracted from water samples. eDNA approach also revealed to be sensitive by detecting 30 supplementary species known as present before the dam construction but never caught by gillnets during 3 years. Furthermore, potential of the marker-genes study might be underestimated since it was not possible to assign some sequences at lower taxonomic levels. Although 121 sequences were generated for this study, a third of species in the area, that exhibits high endemism, are still unknown in DNA databases. Efforts to complete local reference libraries must continue to improve the taxonomic assignment quality when using the non-invasive and promising eDNA approach. These results are of broader interest because of increasing number of hydropower projects in the Mekong Basin. They reveal the crucial importance to sample tissues/DNA of species before dam projects, i.e. before the species could become endangered and difficult to catch, to obtain more precise biomonitoring in the future as we believe eDNA metabarcoding will rapidly be integrated as a standard tool in such studies.

## Introduction

Among the anthropogenic impacts on freshwater ecosystems, the possible social and environmental effects of dam, including on food resources, are under scrutiny as numerous hydropower dams are planned [1–4]. The need for electricity and development of alternatives to fossil and nuclear sources of energy for sustainable energy production argue to improve hydroelectricity [5]. However, hydropower reservoirs, the artificial lakes associated with dams construction, are recognized to modify local ecosystems by disturbing the river flows. Reservoir impoundment leads to characteristics change of water, either locally [6] or further downstream [7]. The reservoir also affects water velocity and transforms lotic environments, characterized by flowing waters, to lentic habitats, characterized by relatively still water. This change of ecosystem constitutes a major adaptive challenge for resident fish species to breed, feed and protect themselves from predators.

A major concern of large reservoirs creation is their potential effects on biodiversity [4]. The habitat fragmentation generated by dams can induce isolation of fish populations [8]. Fish populations being not connected anymore, a reduction of genetic diversity and a decrease of effective population size may be observed for some species. Genetic structure can be significantly modified as well [9]. This is particularly true for migratory species that show an additional risk of stochastic extinctions [10]. Finally, although a short-term increase in fish biodiversity is commonly observed in newly impounded reservoir (mixture of species) during the trophic upsurge period [11], it is generally followed by a decrease in biodiversity in the long-term [1]. Nevertheless, this observation is not systematically true for all dams [12].

The Mekong River is the world’s 10^th^ longest river, extending almost 4,900 km from the Tibetan Plateau in China to its mouth in southern Vietnam. Its physical diversity, tropical location, and high productivity have fostered the evolution of a diverse fish community of about 850 freshwater species [13]. As a result, the Mekong basin is recognized to host one of the highest fish diversity. It is also a river particularly concerned by the effects of dam constructions on the mainstream or tributaries [4]. Up to 135 fish species evolved migrating life history strategies, making them potentially very sensitive to dam constructions as predicted by modelling [3].

Unfortunately, few studies are available to evaluate over time the effects of dams/reservoirs on biodiversity in the Mekong area. In particular, more accurate detection and record of species are necessary to improve management. When fish species are or become rare, classical field monitoring techniques may not allow to detect them properly. However, new non-invasive methods have been recently proposed to evaluate biodiversity relying on DNA present in the environment [14]. By targeting specifically the DNA of one species of interest, a positive PCR or qPCR amplification obtained from the environmental DNA (eDNA) sample will detect the presence of this species in the environment even if no individual is observed directly [15,16]. Moreover, DNA disappearing rapidly in freshwater, the DNA detection is almost contemporary with presence of the species [17]. Alternatively, bulk detection of species can also be performed by amplifying eDNA by PCR with universal primers from discriminating markers (e.g. mitochondrial ones for animals or chloroplast or ITS genes for plants) and by sequencing the PCR products in depth by next-generation sequencing technologies [14,18,19]. This latter method, known as metabarcoding, is more efficient and cost-effective to obtain information on a larger scale. A major advantage is that no species are targeted specifically, which makes possible to highlight the presence of unexpected species. The eDNA metabarcoding approach appears to be more sensitive [16] than traditional fishing study and shows promise in various aquatic environments [19], with faithful description of local fish biodiversiy along a river [20]. However, pitfalls remain concerning reliability [21], and false positives have been reported for headwater stream [22].

The choice of markers targeted for such studies is of primary importance. Although the cytochrome c oxidase subunit 1 (COI) is a marker of choice for single animal specimen DNA barcoding (see BOLD, [23]), its interest has been disputed for DNA metabarcoding studies [24]. Primer binding sites within this gene are not highly conserved and the amplification of fish using a single primer pair is not possible [25]. In addition, the length of the fragment usually targeted is long (>500bp). It is not suitable for eDNA metabarcoding studies because DNA is susceptible to degradation in the environment and fragments are usually shorter [14,26,27]. Other mitochondrial markers have thus been explored to identify animals and the amplicon size has been reduced [28]. In addition, recent studies have shown the interest to simultaneously analyse multiple markers to improve the probability of species detection [24,29–31]. Considering these observations, two different mitochondrial genetic markers were retained to monitor fish species for this survey: (i) a short 12S fragment (100pb) for which the primers were designed and tested *in silico* [27]. This fragment is proved to be reliable on degraded DNA [29], is informative to discriminate vertebrates species [27] and is adapted to survey fish diversity (e.g. [30]); (ii) a longer cytochrome *b* fragment (235bp) to potentially improve the taxonomic resolution [26]. This fragment was designed and used for the surveillance of fish species composition in freshwater in Asia [32].

The Nam Theun 2 hydropower Reservoir (NT2) area [33] was selected for this survey. Numerous villages are settled along the reservoir [34] and the two rivers downstream that are the Nam Theun River (NTH) and the Xe Bangfai River (XBF). Fisheries are further recognized as an important source of income for those villages and might be affected by the evolution of fish diversity. To assess changes in fish population in the NT2 reservoir area (Reservoir, River upstream and downstream), a monitoring by surface gillnet is performed since the 2008 and is planned until the end of the Concession Agreement (2035). It makes the NT2 area particularly suitable to evaluate an eDNA metabarcoding approach and to potentially improve the fish species record. A more accurate monitoring of fish species would be useful to better implement resources management for the human populations. Notably, it would help to identify which species could be lost or threatened, including economic valuable species, but also monitor potentially invasive species.

The fish diversity remains far from being well known in over most Asia as very large areas are unsurveyed [35]. Interestingly, this is not the case for the NT2 area. In the framework of the NT2 project, 8 fish biodiversity surveys were conducted by Kottelat between 1996 and 2012. They assessed and described the fish species in the two rivers based on morphological criteria. These surveys led to obtain a well referenced catalogue of fish species for the NTH basin (74 species) and the XBF basin (178 species), mainly recorded before the dam construction [35]. Among them, 54 species were reported for the first time in Lao PDR and 25 new species were described [35]. Fish species present in the NT2 area display two kind of specificities that broadened the interest of this survey: (i) a high endemism in the rivers affected by the reservoir and (ii) a fish diversity typical of Mekong tributaries that is still being inventoried in other rivers of the area [36].

During 3 years and through 5 campaigns (2014–2016), an experimental surface gillnet study combined to an eDNA survey were conducted on 8 different sampling sites. Fish captured were morphologically assigned to a species while a non-invasive approach was conducted from DNA extracted from water samples to identify which taxonomic groups were present, ideally up to the species level. The objectives of this survey were methodological first with the intent of improving the available tools to better evaluate biodiversity for future monitoring. The main aims of the assay were to (i) implement and refine the method to record the fish species in the NT2 area by including analyses of two marker genes amplified from environmental DNA, (ii) evaluate difficulties, benefits and complementarity of each method in a context of high endemism and lack of species information (e.g. DNA), (iii) improve the reference sequence databases by sequencing local taxa poorly or not yet represented to enhance the quality of taxonomic assignments, (iv) develop a tested multidisciplinary approach to assess fish biodiversity for further potential use of the tool in a context of reservoir projects in the Mekong basin.

## Materials and methods

### Ethics statement

Fish sample and monitoring were performed by the Nam Theun 2 Power Company who has the national authorization for fish biodiversity monitoring and research in rivers and reservoir by the Lao Governement namely the Ministry of Agriculture and Forestry representative on Province level and the Nakai Natural Protected area (namely the Watershed Management Protection Secretariat). This authorization includes the fishing method that are the gillnets. This authorization started in 2008 to allow a fish biodiversity monitoring in the dam area and as an obligation of monitoring between the company and the Government of Laos in the Concession Agreement—Environment and Biodiversity Obligation.

All our activities were authorized and included all the Nam Theun 2 area (Nam Theun watershed and Xe Bangfai Watershed). We only avoid fishing in the Village Fish Protected Zone in the Xe Bangfai and performed our survey in authorized fishing area (same as local people). All location and activities were authorized as it is a part of the environment and biodiversity monitoring obligation in the concession agreement between the Company and the Governement of Laos.

As stated in the concession agreement between the Company and the Governement of Lao, obligation includes the monitoring of endemic species that could be defined as endangered as species were not known yet. No protected species were involved.

Traditional fishing method (gillnet sampling overnight) was used to collect the fish. Specimen were collected and put in freezer for further analysis. Fish collected were already dead specimen in the gillnets. All fish flesh samples were collected on dead and frozen specimen.

All our fish sampling activities were authorized by the Ministry of Agriculture and Forestry of Laos representative on Province Level. The authorization concerns all the Nam Theun 2 area (Nam Theun watershed and Xe Bangfai Watershed) that includes fish research and namely the sampling procedures.

### Area of study

**Fig 1:**
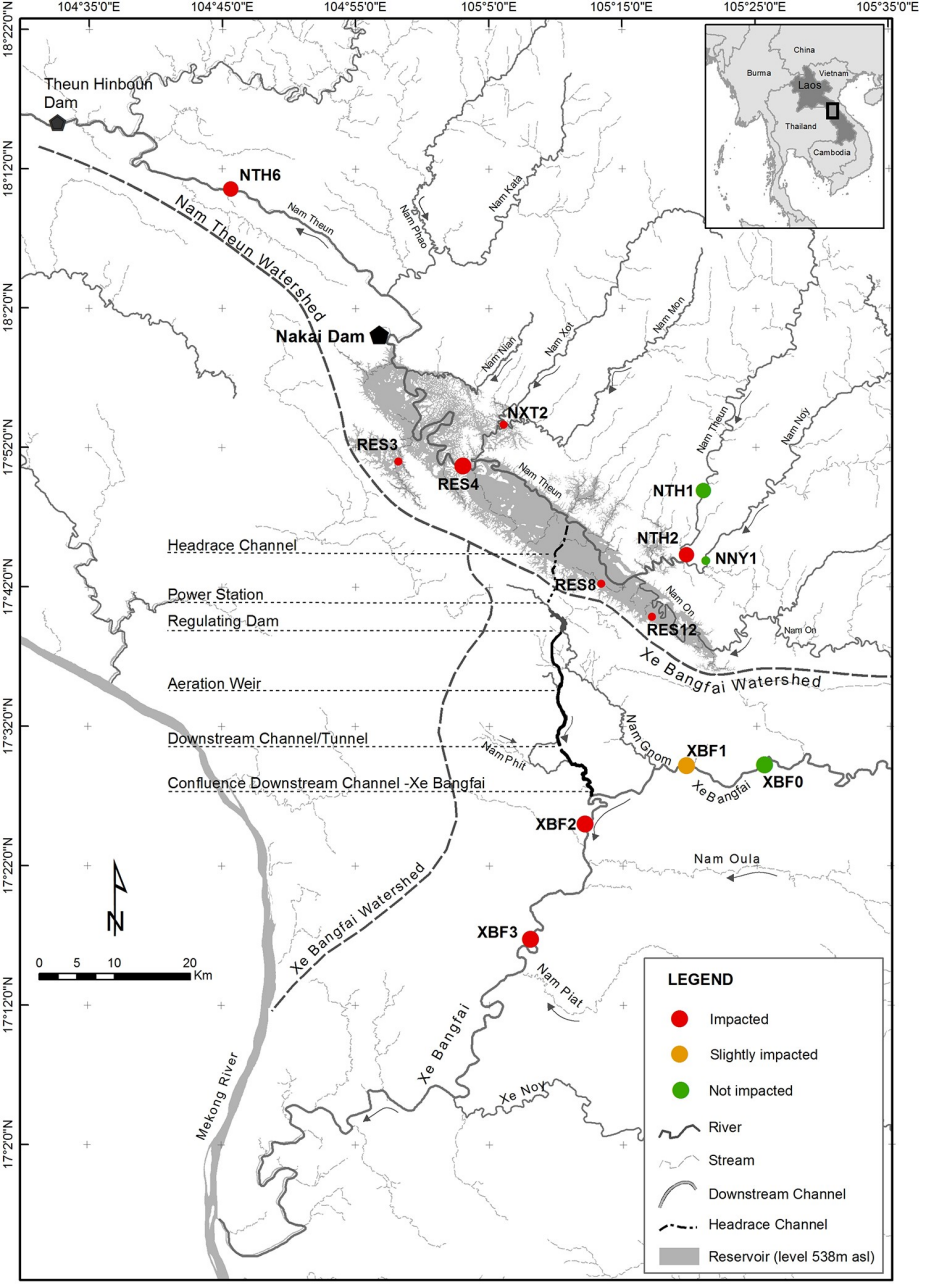
The Nam Theun 2 area with the location of the Nakai dam and the sampling sites used in the study (dots). The dots are colored according to their status: affected, slightly affected or not affected by the dam in terms of water quality features, backwaters, reservoir and fluctuation levels or river flow. The larger dots correspond to the sites sampled for eDNA metabarcoding monitoring (see text and S1 Table for geographic coordinates).

The Nam Theun 2 hydropower Reservoir (NT2) is located in the Middle Mekong Basin, on the Nam Theun River (NTH), a tributary of the Mekong, in Khammouan Province in central Lao PDR. The reservoir was impounded in 2008 and the commercial operation, supervised by the Nam Theun 2 Power Company (NTPC), started in April 2010. The water intake, situated on the middle part of the reservoir on the Nakai Plateau, conducts the water through a headrace and pressure tunnel to the power station. The turbinated waters are diverted from the NTH watershed to the Xe Bangfai River (XBF) watershed by an artificial downstream channel (DSC) (Fig 1). All features are available and were detailed previously [33]. The NT2 Reservoir is qualified as shallow (average depth of 8m; [37]) and its surface fluctuates from 489 km^2^ to 86 km^2^, at its full supply level and at the minimum operation level respectively.

The sampling sites in the NT2 area were located along the NTH river upstream (NTH1) and downstream (NTH6) of the reservoir, in the transition area between the river and the reservoir (NTH2), and on the reservoir (RES4). Sampling sites along the XBF river were located upstream (XFB0 and XBF1) and downstream (XBF2 and XBF3) of the release of turbinated waters (Fig 1, S1 Table for more detailed information).

### Fish monitoring

Since 2008, a fish population monitoring was carried out twice a year [12] at the end of the warm-dry (WD) season (March to May) and at the end to the warm-wet (WW) season (September to November). Surface gillnets with different mesh size (total fishing surface of 1000m^2^) were set overnight at each site. The fish collected were morphologically identified at the species level after each campaign [12] based on the taxonomic keys of [38–40]. In order to compare more easily the species names with the marker gene study, the nomenclature was unified by using by default the NCBI taxonomy when available. Indeed, some species are known by different names (e.g. *Hypsibarbus vernayi* is referenced with 4 other synonym names, but no one is from the same genus), making comparison between publications/identifications sometimes difficult. Samples considered for this study were collected during the fish population monitoring conducted in 2014 (WD and WW), 2015 (WD and WW) and 2016 (WD only). The five sampling campaigns were named as C1, C2, C3, C4 and C5 respectively.

For each sampling campaign, small pieces of tissue samples of different fish were collected. A total of 88 species (559 individuals) were sampled during the survey, including sometimes species captured from other stations in the area (identified by smaller dots in Fig 1).

### Water sampling for eDNA study

Since 2014, water was sampled in the 8 sites described above (Fig 1) to perform the eDNA metabarcoding study. For each sampling site, samples of water were collected with a Niskin bottle of 2 litres at 3 different depths (bottom, middle and surface) of the water system (river or reservoir). The 6 litres were mixed together in a tank and filtered at the NTPC Environment Department on Whatman nitrocellulose membranes with a pore size of 0.45 μm (MicroPlus-21) using a filtration system (Nalgene). As much filters as necessary were used to pass all the 6 litres. The filters of a same sample were pooled together and conserved at -20°C before being sent and analysed at the IGFL sequencing platform. In total, 45 samples of pooled filters were analysed for this study as the reservoir was sampled twice.

Before each field sampling process, tanks were successively rinsed during 15 seconds with ethanol 90%, then with ultrapure water. To monitor simultaneously possible contamination of the ultrapure water and efficiency of tanks decontamination process, 6 litres were filtered for each campaign of sampling in the same process of samples. They constituted 5 water-blank controls that were included to the samples for analysis.

### Cautions and controls for the metabarcoding study

DNA extraction, PCR set-up and post-PCR analysis were conducted in three different dedicated and physically isolated IGFL facilities to prevent samples from DNA contamination (Supplementary Information, SI). Controls were performed during DNA extraction and PCR amplification steps to monitor possible DNA contamination of reagents, equipment and lab areas. An extraction blank control, using only buffer without adding sample, was included in each extraction session. Different negative controls were similarly completed at each PCR session and replicates were done (at least two independent PCR sessions with at least two replicates by sample and session). Because the samples were distributed in two campaigns by year, all the experiments were spaced in time, reducing the possibility to have cross-contamination between sampling campaigns.

### DNA extraction

After different preliminary tests (SI), Nucleospin Soil kit (Macherey Nagel) was used with a combination of Lysis Buffer SL1 and Enhancer SX. For each sample, frozen filters were crushed using a scalpel and incubated overnight at 37°C with 5ml of SL1 buffer under gentle agitation. Starting from 1.4ml, the DNA extraction was performed according to the manufacturer’s recommendations (detailed protocol is given in SI).

### Fusion primers and blocking primers design

For this study, a PCR approach targeting short fragments of two mitochondrial markers (12S RNA and cytochrome *b*, hereafter 12S and cyt*b* respectively, Table 1) was designed. Blocking primers, identified as a better approach than restriction enzymes [41], were also added to prevent the amplification of human DNA that is a recurrent contaminant [29]. The 12S-V05_F and 12S-V05_R primers pair described to be vertebrates specific [27] was used to amplify an insert of around 100bp of the 12S. The cyt*b* primers H15149 [42] and L149125 [32], targeting around 235bp, should preferentially allow the amplification of fish species. H15149 was slightly modified (Y instead of C in the 3’ part) to take into account the cyt*b* diversity observed for sequences of local fish known at the beginning of the project.

**Table 1:** Primers used for this study.

	Primer Name	Primer sequence 5'-3'	References	Insert length
**Cyt*b***	Fish-CytB-H15149	GGTGGCKCCTCAGAAGGACATTTGKCCYCA	Modified from (42)	235bp
	Fish-CytB-L14912	TTCCTAGCCATACAYTAYAC	(32)	
	CytB-L14912-Blocking-Human	TAGCCATGCACTACTCACCAGACGCC-SpacerC3	This study	
	FishCytB-L14912-FusionA-BC1	CCATCTCATCCCTGCGTGTCTCCGACTCAG-CTAAGGTAACGAT-TTCCTAGCCATACAYTAYAC	This study (Ion Torrent A adaptor + barcodes + primer)	
	FishCytB-L14912-FusionA-BC2	CCATCTCATCCCTGCGTGTCTCCGACTCAG-TAAGGAGAACGAT-TTCCTAGCCATACAYTAYAC		
	FishCytB-L14912-FusionA-BC3	CCATCTCATCCCTGCGTGTCTCCGACTCAG-AAGAGGATTCGAT-TTCCTAGCCATACAYTAYAC		
	FishCytB-L14912-FusionA-BC4	CCATCTCATCCCTGCGTGTCTCCGACTCAG-TACCAAGATCGAT-TTCCTAGCCATACAYTAYAC		
	FishCytB-L14912-FusionA-BC5	CCATCTCATCCCTGCGTGTCTCCGACTCAG-CAGAAGGAACGAT-TTCCTAGCCATACAYTAYAC		
	FishCytB-L14912-FusionA-BC6	CCATCTCATCCCTGCGTGTCTCCGACTCAG-CTGCAAGTTCGAT-TTCCTAGCCATACAYTAYAC		
	FishCytB-L14912-FusionA-BC7	CCATCTCATCCCTGCGTGTCTCCGACTCAG-TTCGTGATTCGAT-TTCCTAGCCATACAYTAYAC		
	FishCytB-L14912-FusionA-BC8	CCATCTCATCCCTGCGTGTCTCCGACTCAG-TTCCGATAACGAT-TTCCTAGCCATACAYTAYAC		
	FishCytB-L14912-FusionA-BC9	CCATCTCATCCCTGCGTGTCTCCGACTCAG-TGAGCGGAACGAT-TTCCTAGCCATACAYTAYAC		
	FishCytB-L14912-FusionA-BC10	CCATCTCATCCCTGCGTGTCTCCGACTCAG-CTGACCGAACGAT-TTCCTAGCCATACAYTAYAC		
	FishCytB-L14912-FusionA-BC11	CCATCTCATCCCTGCGTGTCTCCGACTCAG-TCCTCGAATCGAT-TTCCTAGCCATACAYTAYAC		
	FishCytB-L14912-FusionA-BC12	CCATCTCATCCCTGCGTGTCTCCGACTCAG-TAGGTGGTTCGAT-TTCCTAGCCATACAYTAYAC		
	FishCytB-H15149-FusionP1	CCTCTCTATGGGCAGTCGGTGAT-GGTGGCKCCTCAGAAGGACATTTGKCCYCA	This study (Ion P1 adaptor+primer)	
**12S**	Vert-12SV5-F	TTAGATACCCCACTATGC	(27)	100bp
	Vert-12SV5-R	TAGAACAGGCTCCTCTAG	(27)	
	HumBlock-12S-HomoB	CTATGCTTAGCCCTAAACCTCAACAGTTAAATCAACAAAACTGCT-SpacerC3	(29)	
	Vert12SV5F-FusionA-BC1	CCATCTCATCCCTGCGTGTCTCCGACTCAG-CTAAGGTAACGAT-TTAGATACCCCACTATGC	This study (Ion Torrent A adaptor + barcodes + primer)	
	Vert12SV5F-FusionA-BC2	CCATCTCATCCCTGCGTGTCTCCGACTCAG-TAAGGAGAACGAT-TTAGATACCCCACTATGC		
	Vert12SV5F-FusionA-BC3	CCATCTCATCCCTGCGTGTCTCCGACTCAG-AAGAGGATTCGAT-TTAGATACCCCACTATGC		
	Vert12SV5F-FusionA-BC4	CCATCTCATCCCTGCGTGTCTCCGACTCAG-TACCAAGATCGAT-TTAGATACCCCACTATGC		
	Vert12SV5F-FusionA-BC5	CCATCTCATCCCTGCGTGTCTCCGACTCAG-CAGAAGGAACGAT-TTAGATACCCCACTATGC		
	Vert12SV5F-FusionA-BC6	CCATCTCATCCCTGCGTGTCTCCGACTCAG-CTGCAAGTTCGAT-TTAGATACCCCACTATGC		
	Vert12SV5F-FusionA-BC7	CCATCTCATCCCTGCGTGTCTCCGACTCAG-TTCGTGATTCGAT-TTAGATACCCCACTATGC		
	Vert12SV5F-FusionA-BC8	CCATCTCATCCCTGCGTGTCTCCGACTCAG-TTCCGATAACGAT-TTAGATACCCCACTATGC		
	Vert12SV5F-FusionA-BC9	CCATCTCATCCCTGCGTGTCTCCGACTCAG-TGAGCGGAACGAT-TTAGATACCCCACTATGC		
	Vert12SV5F-FusionA-BC10	CCATCTCATCCCTGCGTGTCTCCGACTCAG-CTGACCGAACGAT-TTAGATACCCCACTATGC		
	Vert12SV5F-FusionA-BC11	CCATCTCATCCCTGCGTGTCTCCGACTCAG-TCCTCGAATCGAT-TTAGATACCCCACTATGC		
	Vert12SV5F-FusionA-BC12	CCATCTCATCCCTGCGTGTCTCCGACTCAG-TAGGTGGTTCGAT-TTAGATACCCCACTATGC		
	Vert12SV5R-FusionP1	CCTCTCTATGGGCAGTCGGTGAT-TAGAACAGGCTCCTCTAG	This study (Ion P1 adaptor+primer)	

### Libraries construction and sequencing

To reduce manipulation steps and cost, barcoded fusion primers were used to perform the PCR amplification and to construct the Ion Torrent libraries in a single step. Each universal forward primer was modified by the addition of the A adaptor sequence and by one specific barcode (12 different barcodes were used for this study) on the 5’ end. The reverse primer was fused in 5’ with P1 adapter sequence (Table 1). By this way, barcoded PCR products can be pooled and purified to eliminate primer dimers (when needed), and sequenced using an Ion Torrent PGM sequencing platform (Thermofisher) without any further library construction steps.

All DNA amplifications were carried out in a final volume of 25μl, using Taq environmental Master Mix 2.0 (Thermofisher), 0.4μM of each primer (Table 1), 4μM of human blocking primer and 2μl of DNA extract (template) or ultra-pure water for negative controls. The PCR program was as follows: 10min at 94°C, 50 cycles of 30s at 95°C, 1 min at 55°C (12S) or at 50°C (cyt*b*) and 30s at 72°C, final elongation during 7min at 72°C.

The same barcode was attributed to a given sample. For each marker, amplicons were pooled all together in an equimolar manner and protocols were followed to remove remaining adapter dimers (SI). Then, the 12S and cyt*b* pooled libraries were equally mixed and diluted to 10pM for emulsion PCR. Template preparation procedure and sequencing followed the Ion PGM standard protocols from Thermofisher (Ion PGM Hi-Q OT2 and Ion PGM Hi-Q Sequencing Kits). At most 12 samples were sequenced on a single run. Eight runs on 318v2 chips were done for this project and sequenced on Ion Torrent PGM.

### Building of reference sequences databases

The ecoPCR program [26,43] was fed with the primer sequences of both markers (Table 1) to construct a reference database for the 12S and cyt*b* fragments using all mitochondrial sequences retrieved from Genbank (release 212). Different values of parameters (i.e. e, maximum number of mismatches; minimum (l) or maximum (L) length of the in silico amplified fragment) were tested and the following ones were finally retained: 12S (e = 3, l = 50, L = 150) and cyt*b* (e = 6, l = 150, L = 300). The final size of each reference database was of 13,476 and 75,862 sequences for 12S and cyt*b* respectively. From surface gillnet captures and morphologically assigned fish individuals, 62 sequences of 12S and 59 sequences of cyt*b* were generated for this study in order to improve the number of representatives of endemic fish species of the NT2 area, mostly unknown in molecular databases. These new sequences were obtained performing the experiments detailed in SI and finally added to the reference databases (Genbank accession numbers: MH688181-MH688301).

### Taxonomic assignment of reads

OBITools 1.2.9 [44] is a free-available set of python programs (https://pythonhosted.org/OBITools/welcome.html) specifically designed for analysing NGS data in a DNA metabarcoding context, in particular for biodiversity surveys from eDNA. Compared to packages for similar purposes like MOTHUR [45] or QIIME [46], OBITools relies on filtering and sorting algorithms, ultimately allowing to associate a taxonomic information with each sequence record. The OBITools package was demonstrated useful in many recent publications and was used for this survey. In a first analysis, the runs corresponding to filters of C1 and C2 sampling campaigns and two different protocols of DNA extraction were analysed together. In a second analysis, the runs corresponding to the filters of the 5 sampling campaigns (C1, C2, C3, C4 and C5) treated with the second DNA extraction protocol, were analysed together.

Roughly the analyses were conducted as follow. In a preliminary step, the fastq files for all the runs were cleaned (keeping only reads with 90% of bases > Q20 with the Filter_by_quality tool of Galaxy, FASTX-Toolkit, A. Gordon). Then, the files were treated with a succession of OBITools programs. First, the reads for each run were sorted and assigned to samples using barcodes and to markers (12S or cyt*b*) with the *ngsfilter* program (using the–t option to specify the file containing the samples description). Then, for each marker, files of runs to consider for analysis were concatenated. The *obiuniq* program was then used to group together identical reads. The number of times each read was observed and in which sample (-m option) are then registered in the new sequence name as attributes. Only sequences for which counts where > or equal to 5 and for which length was comprised between 50bp and 150 bp (12S) or 150bp and 300bp (cyt*b*) were kept with the *obigrep* program. This last file was then cleaned for PCR and sequencing errors using the *obiclean* program. Finally, the *ecotag* program was used to assign sequences to taxa by using the reference databases built for 12S and cyt*b* (see above).

R-scripts and STAMP [47] were used to finalize the statistical analyses of the data. STAMP is a software package for analysing taxonomic profiles and providing statistical tests. In particular, its graphical interface allows an easy exploration of the data.

Because the number of total reads vary between samples (a sample is considered here as one genetic marker for one given site and one given sampling campaign, or a control), the reads counts assigned by taxa were converted in percentage by sample. Before computing this percentage, all taxa for which less than 5 reads were obtained were removed from each sample in order to avoid possible artefacts. This threshold was arbitrary chosen in absence of consensus in the community [48], even if removing singletons or doubletons at least is quite usual.

### Intragroup percentage of pairwise sequence differences

The genera of 71 fish species found in the NT2 area by surface gillnet capture were considered. When known, all sequences corresponding to each genus were retrieved from the reference sequences databases (12S or cyt*b*). The percentage of pairwise differences between sequences of the same taxonomic group at different taxonomic level (species, genus, family) were computed with MEGA7 [49]. This allowed us to choose a threshold of assignment to the species level.

## Results

### Fish diversity observed by surface gillnet method

Morphological identification of fish captured by surface gillnets allows to identify a total of 11 orders and 93 species in the NT2 area (Table 2; S2 Table for detailed dataset). Cypriniformes and Siluriformes are the most represented orders with respectively 62 and 15 species. The remaining orders are all represented by less than 5 species, and by a single species in 5 cases out of 8.

**Table 2:** Fish identified by surface gillnet capture in the Nam Theun area during five sampling campaigns (C1 to C5) and for the 8 sites sampled for eDNA metabarcoding (4 on NTH and 4 on XBF).

Taxonomic levels identified by surface gillnet capture				Fish frequencies (%)	
Order	Family	Genus	Species	NTH	XBF
Anabantiformes	Anabantidae	1	1	0.52	0
	Channidae	1	1	0.22	0.10
	Pristolepididae	2	2	0	0.78
Beloniformes	Belonidae	1	1	0	4.00
Cichliformes	Cichlidae	1	2	1.04	0
Cypriniformes	Cobitidae	3	4	0	1.14
	Cyprinidae	35	57	90.94	68.93
	Gyrinocheilidae	1	1	0.13	0
Gobiiformes	Eleotridae	1	1	0.18	0
Osteoglossiformes	Notopteridae	2	3	0	1.92
Perciformes (?)	Ambassidae*	1	1	5.80	2.55
Pleuronectiformes	Soleidae*	1	1	0	0.23
Siluriformes	Bagridae	3	5	0	2.71
	Clariidae	1	1	0.53	0
	Pangasiidae	1	3	0	4.47
	Schilbeidae	2	2	0	6.99
	Siluridae	3	4	0	5.25
Synbranchiformes	Mastacembelidae	2	2	0.64	0.77
Tetraodontiformes	Tetraodontidae	1	1	0	0.16
**Total 11**	**19**	**62**	**93**	**100%**	**100%**

Only order and family names are indicated with the corresponding number of genera and species identified morphologically. The mean frequencies of fish observed (computed from number of individuals) across the 5 campaigns are given in percentage for the Nam Theun River sites (NTH) and the Xe Bangfai River sites (XBF). The total line gives the number of taxonomic groups assigned at different level.

* indicates taxa not found by eDNA. (?) The classification of this species is questioned.

The distribution through time shows that 6 orders are detected in all sampling campaigns (S2 Table): Cypriniformes, Siluriformes, Perciformes, Anabantiformes, Beloniformes and Cichliformes. The others are detected only one year (e.g. Tetraodontiformes in C1 and C2, Gobiiformes in C2 and C3) or during the WD season (Synbranchiformes, e.g. *Macrognathus siamensis* in the XBF). Some species appear preferentially in a single site, as for example the Tetraodontiformes *Auriglobus nefastus* in XBF downstream (XBF3), the Pleuronectiformes *Brachirus harmandi* in XBF upstream (XBF0) or the Gobiiformes *Oxyeleotris marmorata* in NTH downstream (NTH6). At contrary, 25 species constitute a “core” group detected in all 5 sampling campaigns, regardless sites (S2 Table). This group is formed by a single representative of Belonidae, Cichlidae, Ambassidae, Bagridae, Claridae, Schilbeidae, Siluridae and of 18 species of Cyprinidae. Finally, 2 species have been detected in each sampling site at least in one sampling campaign and have been found systematically in every sampling campaign in at least one site: *Hypsibarbus wetmorei* and *Parambassis siamensis*.

The distribution across sampling sites identifies a strong difference between both the NTH and the XBF in terms of species richness. The NTH is clearly less diverse than the XBF with only 7 orders (30 species) detected for all 5 sampling campaigns taken together instead of 9 orders (78 species). By considering the “core” group species, this observation is even more striking: 11 are present in both rivers, but only 3 are specifically observed in NTH although 11 are specifically found in XBF.

### Controls and identification of vertebrates by eDNA

Controls were performed all along the eDNA metabarcoding study to monitor contamination from the sampling to the sequencing step (SI). No or rare contaminants (human, pig) were detected in extraction and PCR controls. However, sporadic contaminants, usually of a single fish species, was sometimes observed in the water-blank controls. According to their nature or distribution, these sporadic contaminants do not hamper the data analyses or the confidence in the results obtained as detailed in SI. Above all, the strict process followed to perform the experimental analyses has been proved to be efficient to track all possible sources of contaminant, a relevant point when universal primers are used.

After removing contaminants, only reads for which a match in the reference databases was obtained with a BLAST identity score higher or equal to 97% and 95% for 12S and cyt*b* respectively were primary considered (SI). Around 8.5M reads were thus kept for analyses with about 5 times more 12S sequences than cyt*b*. Both primer pairs were able to amplify different groups of vertebrates. As expected, sequences from fish were mainly observed (95% of reads) when considering all data together. However, amphibian, mammal, bird and even reptile (squamate) species are also identified in the 5% remaining reads (S3 Table). The geographical origin of those non-fish species detected confirmed that all species are present in the Lao PDR, as for example the bony-headed toad (*Ingerophrynus galeatus*), the Chinese pond heron (*Ardeola bacchus*) or the Asian water monitor (*Varanus salvator*). This was also the case for the vast majority of the fish species evidenced.

### Fish diversity observed by eDNA

By considering the taxonomic assignment conditions mentioned above, a large diversity of fish was observed during the whole survey in terms of identified orders (10), families (25), genera (90) and species (124) for both markers taken together (S2 Table). However, the 12S is able to identify more biodiversity than the cyt*b* (2 orders, 8 families and at least 12 species more), and with a lower frequency of unclassified taxa (Tables 2 and 3).

**Table 3:** Fish identified by cyt*b* from eDNA metabarcoding on the Nam Theun area during five sampling campaigns and for the 8 sites sampled.

Taxonomic levels identified with eDNA metabarcoding (cyt*b*)				Fish frequencies (%)	
Order	Family	Genus	Species	NTH	XBF
Beloniformes	Belonidae	1	(1)	0	<0.01
Cichliformes	Cichlidae	2(1)	2	7.31	1.52
			(3)	19.26	1.84
Clupeiformes	Clupeidae	1	1	0	<0.001
Cypriniformes	Cobitidae	4	5	<0.01	1.47
		1(1)	(2)	0	1.73
	Cyprinidae	33(1)	40	24.73	25.97
			(15)	41.39	51.27
	Gyrinocheilidae	1	1	1.90	0.26
			(1)	<0.01	<0.01
	Nemacheilidae	1	1	0	0.03
Osteoglossiformes	Notopteridae	2	2	0	0.47
Siluriformes	Amblycipitidae*	1	1	0	<0.01
	Bagridae	4(1)	6	0	4.07
			(2)	3.93	5.72
	Clariidae	1	2	0.09	<0.01
		(1)	(1)	<0.01	0
	Pangasiidae	1(1)	2	0	0.06
			(1)	0	0.04
	Schilbeidae	2	2	0	1.82
			(1)	0	0.03
	Siluridae	4	4	0	0.65
		2	(2)	0.04	0.02
Synbranchiformes	Mastacembelidae	1	1	0	0.64
			(1)	1.33	2.27
Tetraodontiformes	Tetraodontidae	1	1	0	0.07
		(1)	(1)	0	0.02
**Total 8**	**16**	**63(7)**	**71(31)**	**34(66)**	**37(63)**

Only order and family names are indicated with the corresponding number of genera and species identified using cyt*b* reads (BLAST identity > 95%). The mean frequencies of fish detected (estimated from reads number) across the 5 sampling campaigns are given in percentage for the Nam Theun River (NTH, 4 sites) and the Xe Bangfai River sites (XBF, 4 sites) considering family assignment. The lines in white correspond to data identified at species level and in grey to unclassified species. The total line gives the number of taxonomic groups assigned at different level with additional “unclassified” groups given between parentheses.

* indicates taxon not found with 12S (Table 4)

Despite the difference in taxa richness revealed between both markers, the global results are quite congruent. For instance, all orders identified by cyt*b* are found with 12S (Beloniformes, Cichliformes, Clupeiformes, Cypriniformes, Osteoglossiformes, Siluriformes, Synbranchiformes and Tetraodontiformes). 15 out of the 16 families detected by cyt*b* are also detected by 12S. However, this stands true for only 2/3 of genera evidenced (42 out of the 63). Checking the cyt*b* sequences identified with low BLAST score permitted to evidence 2 more families in common with 12S (Balitoridae and Sisoridae) but not the 2 following missing orders: Anabantiformes and Gobiiformes.

Cypriniformes and Siluriformes are the two orders represented by the larger number of species identified in both markers (respectively 57 and 14 for 12S, 46 and 17 for cyt*b*; Tables 3 and 4). All the remaining orders are represented by less than 4 species, and often by a single one. However, the real number of taxa detected by eDNA metabarcoding is surely underestimated according to the restrictive criteria used to analyse the data.

**Table 4:** Fish identified by 12S from eDNA metabarcoding on the Nam Theun area during five sampling campaigns and for the 8 sites sampled.

Taxonomic levels identified with eDNA metabarcoding (12S)				Fish frequencies (%)	
Order	Family	Genus	Species	NTH	XBF
Anabantiformes*	Anabantidae*	1	1	0.08	0
	Channidae*	1	1	0.01	0.04
	Osphronemidae*	1	(1)	0	0.03
	Pristolepididae*	1	1	<0.01	1.15
Beloniformes	Belonidae	1	1	<0.01	3.38
Cichliformes	Cichlidae	(1)	(1)	0.58	0.12
Clupeiformes	Clupeidae	1	1	0	0.19
		1	(1)	0	0.15
Cypriniformes	Balitoridae*	(1)	(1)	0.01	0.03
	Cobitidae	6(1)	7	<0.01	1.09
			(4)	<0.01	2.98
	Cyprinidae	36(1)	48	53.60	29.90
			(12)	40.28	41.89
	Gyrinocheilidae	1	1	0.50	0.14
	Nemacheilidae	1	1	0.31	0.01
		(1)	(1)	0.23	0.23
	unclassified	(1)	(1)	0.30	1.04
Gobiiformes*	Eleotridae*	1	1	0.05	0
	Gobiidae*	1	1	<0.01	0
Osteoglossiformes	Notopteridae	1	1	0	0.61
		(1)	(1)	<0.01	0.72
Siluriformes	Bagridae	3(1)	4	<0.01	2.29
			(3)	1.86	1.08
	Clariidae	1	1	0.47	6.24
			(1)	0	0.08
	Heteropneustidae*	1	1	0	<0.01
	Pangasiidae	1	3	<0.01	3.20
			(1)	<0.01	<0.01
	Schilbeidae	2	2	<0.01	0.36
		(1)	(1)	0	0.01
	Siluridae	2	2	0.21	0.55
	Sisoridae*	1(1)	1	0	0.01
			(2)	0.09	0.24
	Unclassified	(1)	(1)	0.02	0.14
Synbranchiformes	Mastacembelidae	2	2	1.20	1.46
			(1)	0.18	0.22
Tetraodontiformes	Tetraodontidae	1	1	0	0.32
		(1)	(1)	0	0.09
**Total 10**	**24(2)**	**68(12)**	**82(34)**	**56.5(43.5)**	**50.9(49.1)**

Only order and family names are indicated with the corresponding number of genera and species identified using 12S reads (BLAST identity > 97%).

* indicates taxa not found with cyt*b* (Table 3). See legend Table 3 for other details.

**Fig 2:**
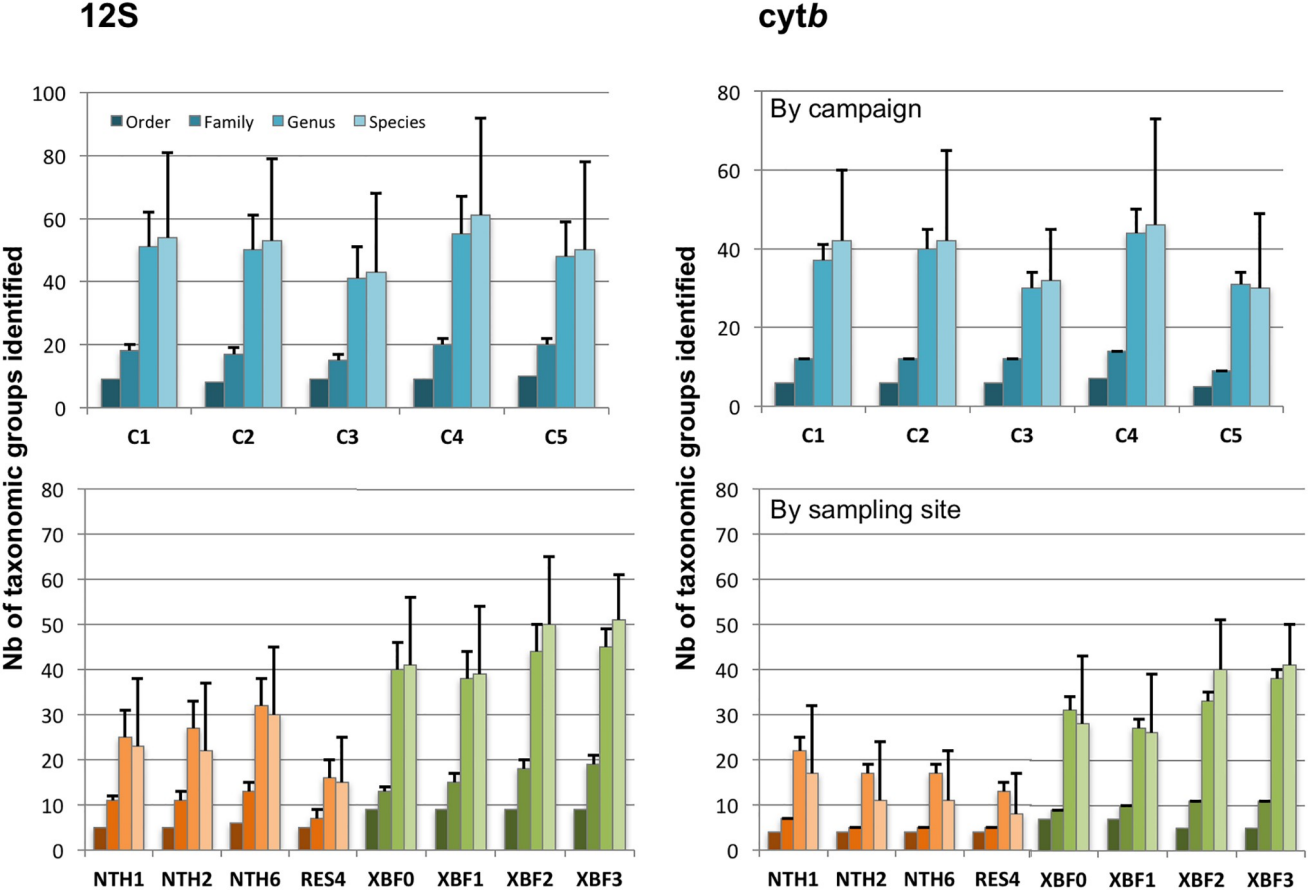
Variation of the number of taxonomic groups identified at 4 levels (order, family, genus and species) for (A) the 12S and (B) the cyt*b*. These numbers are given according to the sampling campaigns (upper part: C1 to C5, see text) or to the sites sampled (lower part: in orange, the 4 sites from the NTH; in green, the 4 sites from the XBF; see Fig 1 for their location on the map). The error bar indicates the putative number of taxa that could be added since it was not possible to identify all taxa precisely (unclassified reads). C1, C3 and C5 are warm dry (WD) season. C2 and C4 are warm wet (WW) season.

Over time, the taxa richness revealed by the two markers appears relatively constant all along the sampling campaigns (Fig 2). Although the taxonomic assignment at a species level should be taken with caution, 16 and 26 species (identified by cyt*b* and 12S respectively) seem present in all sampling campaigns (S2 Table). Among them, 7 species are found by both markers: *Hampala macrolepidota*, *Hemibagrus spilopterus*, *Hypsibarbus vernayi*, *Laides longibarbis*, *Mystacoleucus marginatus*, *Pseudomystus siamensis* and *Raiamas guttatus*. When checking for a possible effect of the season on the taxa richness observed, no significant mean difference of taxa number between WW and WD sampling campaigns is detected. However, the number of genera and species is usually higher for WW (C2 and C4) compared to WD (C1, C3 and C5; Fig 2). Also to note, 3 species, detected in XBF only, could have a season specific signature according to the 12S study. 2 other species, but evidenced by the cyt*b* survey this time, are in a similar situation. Those results have to be taken with caution and would have to be confirmed on more data in the future to exclude possible artefacts.

According to the 12S study, 7 species (among which *Hampala macrolepidota*) appear to have a large geographic distribution in the NT2 area. They are detected in all sampling sites, in at least one sampling campaign. These results will have to be confirmed by the next sampling campaigns.

Overall, strong difference in fish diversity is observed between both rivers (Tables 3 and 4; Fig 2). Without counting unclassified reads and according to 12S, the number of orders is restricted to 6 for the NTH with 37 genera represented (3 orders and 26 genera for the cyt*b*). On the contrary, the XBF is more diverse with up to 8 orders detected corresponding to 57 genera (7 orders and 47 genera for cyt*b*). If the 5 sampling campaigns are totalized and the mean numbers of taxa for the 4 NTH sites vs the 4 XBF sites are compared, the XBF shows in general around twice more taxa than the NTH for each taxonomic level considered (all t-tests significant with p<0.01). Although 18 species are detected very regularly in the XBF (in 4 to 5 sampling campaigns and by 12S exclusively), only one species is often observed in the NTH (S2 Table). This observation is reinforced by the cyt*b* survey for 7 of those species: *Rasbora dusonensis*, *Notopterus notopterus*, *Hemibagrus spilopterus*, *Mystus singaringan*, *Pseudomystus siamensis*, *Pseudolais pleurotaenia* and *Laides longibarbis*. Indeed, all these species are detected only in the XBF by this second marker.

Variations in fish diversity between sampling sites are clearly noticed. The reservoir (RES4) is without contest the sampling site showing the lowest number of recorded species (Fig 2). The two sampling sites downstream of the XBF (XBF2 and XBF3), and located after the confluence with the DSC (Fig 1), are those for which the taxonomic richness is the highest.

### Relative fish taxa frequencies evaluated with surface gillnet or eDNA approach

Although these data should be considered with high caution (see Discussion), fish frequencies using the results of the gene markers studies have been attempted to be estimated. The read counts were converted in percentage (SI) and used as a proxy to evaluate the proportions of fish taxa identified. The not assigned reads (generally unclassified at the genus or species levels) were considered to compute the frequencies at higher taxonomic levels because those reads were assigned without ambiguity at the order level and, in almost all cases, at the family level (Tables 3 and 4).

**Fig 3:**
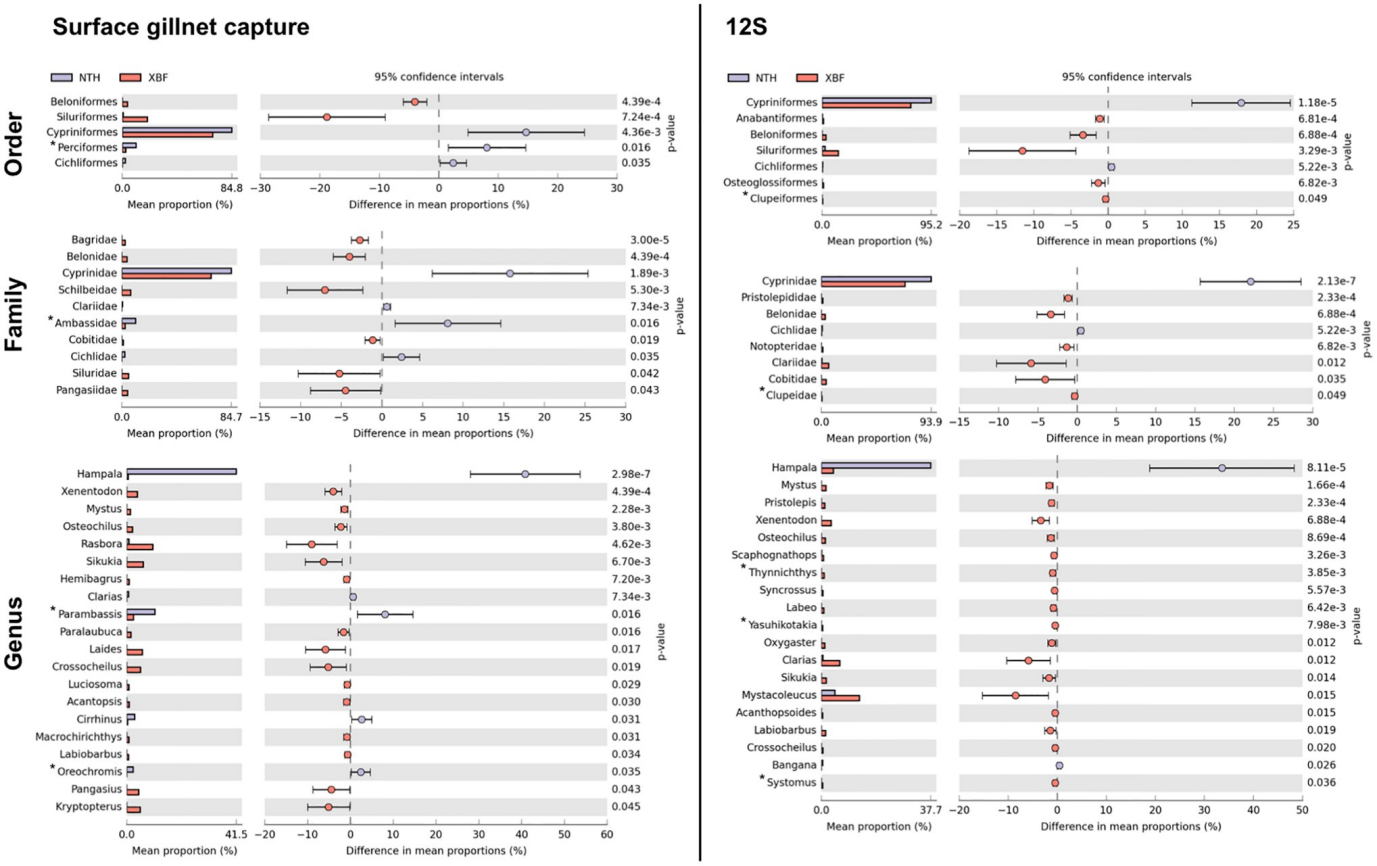
Taxonomic groups showing significant differences (p<0.05) in estimated mean proportions observed between NTH and XBF either by (A) surface gillnet capture or by (B) eDNA approach (12S) during the entire survey. Order, family and genus levels are detailed. Stars indicate groups that are detected by only one method.

Considering the global survey, the 12S approach and the surface gillnet capture reveal similar patterns. Cypriniformes and Siluriformes appear as the most abundant orders in the 2 rivers (Tables 2 and 4). Cypriniformes is by far the most represented order in NT2 area with essentially one main family highly frequent, the Cyprinidae (>90%). Both monitoring methods show that the proportion of Cypriniformes significantly drops in the XBF compared to the NTH. This is notably because the proportion of Siluriformes, nearly absent from the NTH, raises in the XBF (Fig 3). Inside order or family groups, species are not equally represented in proportion, some being rare and others being highly abundant. Thus, *H*. *macrolepidota* appears as the most frequent species with both monitoring approaches. Indeed, this species alone accounts for more than 1/3 of the Cypriniformes representatives in all the NTH sites (Fig 3). This is mainly due to the very high proportion of this species observed in a single sampling site, the reservoir RES4.

Checking the proportions observed for the cyt*b* study, the conclusions are somewhat different. Cypriniformes are still the most abundant order for NTH and XBF (Table 3). However, although Siluriformes are still the second more frequent group for the XBF, Cichliformes are now second in proportion for NTH with this marker. This is mainly due to a single genus, *Oreochromis* genus (also commonly known as Tilapia, Cichlidae), that constitutes for cyt*b* the most abundant taxa in NTH sites, in particular in the reservoir RES4 (more than half in mean for the 5 sampling campaigns).

## Discussion

### Benefits and limits of surface gillnet capture and eDNA approach to assess the species richness in the Nam Theun area

#### On the difficulty sometimes to morphologically assign fish to a given species

The monitoring of fish population using surface gillnets, conducted since 2008 in the NT2 area, provided valuable data in term of taxonomic richness and fish population evolution in a recent reservoir [12] and rivers downstream of a reservoir. A major interest of gillnet survey is that when a species is captured on a given site at a given time, its presence is unequivocal. Its abundance can then be evaluated by the number of individuals that have been fished. However, when the species is rare, it can be difficult to catch. In addition, benthic species have most probability to be missed by surface gillnets. Another difficulty is that fishing requires to morphologically identify the species with precision in a part of the world where the fish diversity is still not well described and have a high level of endemism [35,50]. Fortunately, different inventory surveys conducted by Kottelat were performed to evaluate the fish diversity in the NT2 area before the dam construction and using morphological traits [35,36]. This assessment described 219 species in the XBF and the NTH among which 10% were new for science. 20% of species were also registered for the first time in Laos. Despite precise taxonomic keys, assign individuals with certainty to a given species can be arduous when they are juveniles or when different species share very close traits. In consequence, errors in morphological identification are likely, although difficult to evaluate precisely.

The eventuality of cryptic species [51] present in the NT2 area cannot be ruled out. This phenomenon is widespread among metazoa taxa and biogeographical regions [52] and, as a consequence, to potentially reduce the species richness evaluation when morphological criteria alone are used. DNA is the only way to reveal those species indistinguishable morphologically. In the present survey, 12S and cyt*b* were sequenced in different individuals for species for which no or few genetic data were available before. Preliminary results we obtained for some individuals/taxa in the NT2 area have reached similar conclusions than a recent study [53]. By sequencing the DNA of morphologically assigned fish from the Congo basin, the authors reported undetected taxonomy diversity compared to morphology [53]. In a reverse manner, cases where different species probably correspond to a single lineage were also described [53]. Work is underway in NT2 area to confirm or rebut our preliminary observations on more individuals for the species concerned.

#### Possible explanations for differences observed between 12S and cyt*b* diversity

**Fig 4:**
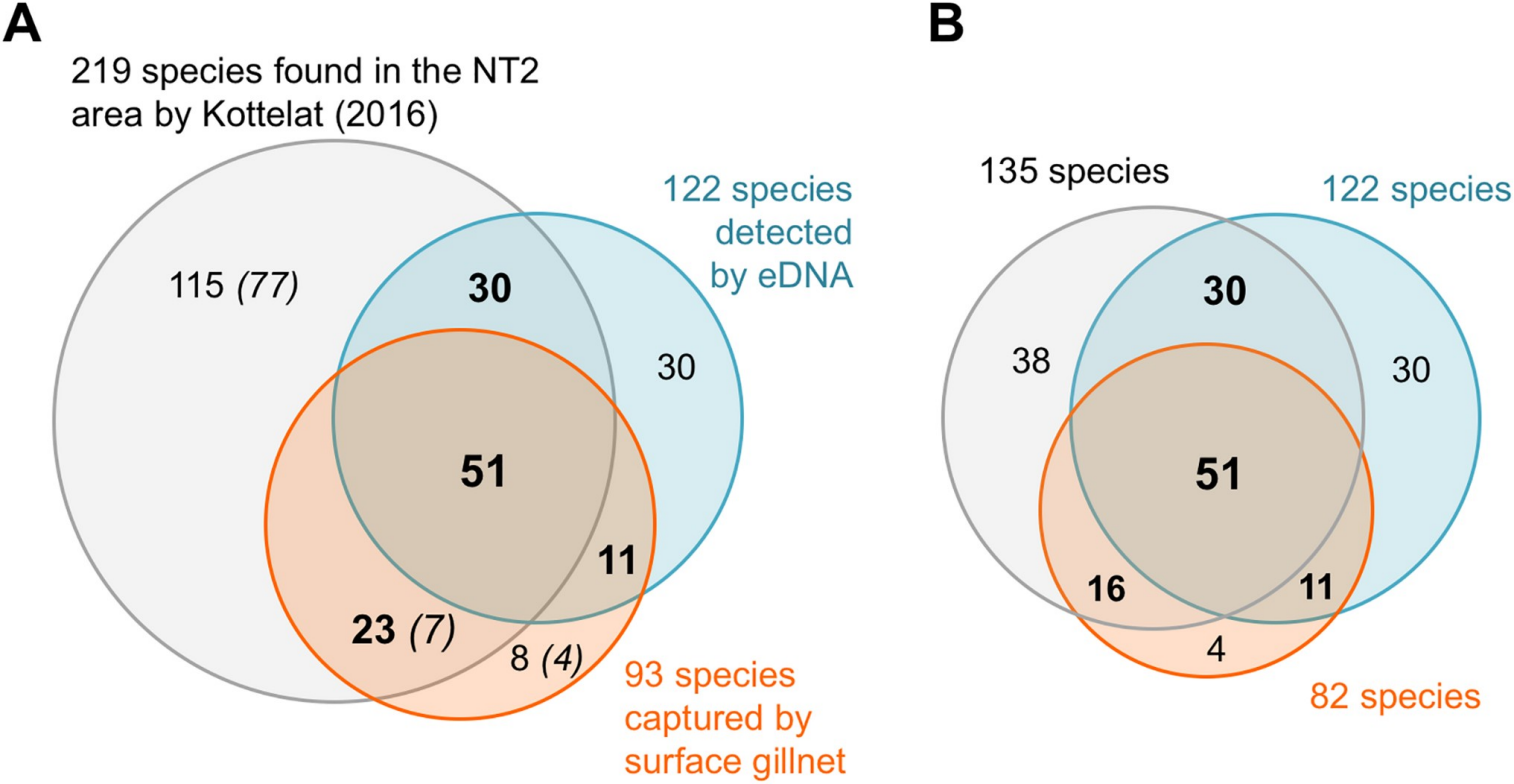
Number of species found in common or exclusively by eDNA metabarcoding or surface gillnet capture in regard to the species described by Kottelat [35] in the NT2 area. (A) all species and (B) restricted to species having a DNA reference in 12S or cyt*b* databases. Only classified species are considered for eDNA and the results of both markers are combined. The numbers in parenthesis in (A) give the species described morphologically but with no DNA reference.

The number of taxa detected and identified by the eDNA metabarcoding approach in this survey (12S and cyt*b* combined) is higher than with the experimental surface gillnets method (Fig 4). This finding is in line with other studies (e.g. [19]). However, differences between markers are observed and notably more diversity is revealed by 12S than cyt*b* (Fig 2, Tables 3 and 4). This can be explained by multiple factors: (i) DNA present in water is rapidly degraded into short fragments. Because the length of the 12S fragment amplified (100bp) is shorter than for the cyt*b* (235bp), more DNA should be accessible and amplifiable with 12S primers, potentially revealing greater diversity; (ii) the read coverage is 5 times higher for the 12S than for the cyt*b*. Although noticeable with 12S, less frequent taxa could thus be not detected with cyt*b* because of the lower sequencing depth. This is potentially explained by the fact that both markers have been pooled to be sequenced together on the same PGM runs. As emulsion PCR preferentially amplifies smaller fragments, a bias of sequencing towards shorter fragments is expected; (iii) the primers used for the 12S are highly conserved throughout the vertebrata [27] and have been extensively used for eDNA metabarcoding studies. On the contrary, the primers designed in the coding gene cyt*b* are degenerated, mainly in third codon position, and less conserved. PCR amplification could be less efficient for some taxa showing more mismatches than usual with the cyt*b* primers and thus, missed. This seems to be the case for *Hampala macrolepidota* for example. Indeed, the *H*. *macrolepidota* cyt*b* sequence shows 3 supplementary mismatches with the cyt*b* reverse primer. It probably explains why this species is not detected at a high frequency for RES4 by the cyt*b* PCR amplification while gillnet capture and 12S identify this species in a large proportion at the same site (Fig 3).

This result alone questioned the reliability of the fish proportion estimates using gene marker studies. Indeed, the quantitative aspect of the eDNA metabarcoding studies is fraught with problems as too many parameters at too many steps can affect these estimates, in particular failure to amplify some species [28] or environmental sampling conditions [54]. If the biomass can be inferred in a quite reliable manner for single species by qPCR for example [55], the primer efficiency is highly species-specific using metabarcoding approach which would prevent accurate assessments of biomass in a sample [56]. In this survey, the relative fish frequencies vary a lot according to the marker used or the species targeted. If the results seem to be roughly indicative with the 12S assay, it is still premature to use them as an efficient and accurate estimate of fish abundance [30]. Moreover, data on populations or individuals, such as biomass, sex, or weight, remain only quantifiable by fishing and direct capture [57].

Despite using degenerate primers, the taxonomic assignment to a species according to the cyt*b* fragment is expected to be more accurate than with the 12S fragment. Actually, the cyt*b* fragment is longer and more variable than the 12S one. Thus, the identification of a species usually relies on more discriminating positions, reducing possible taxonomic assignment errors linked to sequencing or PCR artefacts. The database is also more complete for the cyt*b* (around 5 times more sequences than for 12S). Incorrect taxonomic assignment could thus occur more often with 12S than cyt*b*, probably with the identification of close species when the real one is missing. We thus confirmed that multiple marker essays are crucial, to take benefits from their specific advantages or to circumvent their distinctive problems, to improve the biodiversity report [30,31].

#### What can be learnt from the unclassified sequences for eDNA metabarcoding

Although the dataset was restricted to reads with high BLAST identity to the references, it was not possible to assign a part of sequences to all taxonomic levels (called “unclassified” taxa). As one can expect, the higher the taxonomic level, the lower the unclassified category occurs (Fig 2). Therefore, all sequences can be classified at the order level and virtually all at the family level (with 2 exceptions inside the Siluriformes and Cypriniformes groups for the 12S, Table 4). For both markers, 11% and 18% reads are unclassified at the genus level. However, this percentage reaches 41–45% when the species level is considered (31/71 and 34/82 for cyt*b* and 12S respectively). Different explanations may be suggested: (i) the sequences of close taxa (species/genus) are not variable enough and cannot be distinguished. The taxonomic assignment is then made to the most recent common ancestor; (ii) the species is unknown. The taxonomic assignment could be done to the upper level (genus or family) if close species/genus are available in the reference database. Note that a wrong species assignment is also possible if a single taxa is referenced for the whole genus/family and the BLAST identity score is high; (iii) sequence errors could interfere with taxonomic assignment, even after data cleaning and artefact removing. It would possibly increase artificially the number of unclassified reads.

Overall, the unclassified data suggest that at least 53 species not yet identified could be present in NT2. This result is not unexpected as the reference databases are not complete. In particular, although 62 and 59 new sequences of 12S and cyt*b* respectively were provided for this study, reference sequences of 88 species described with morphological criteria in the NTH and the XBF are still missing for both markers. Nevertheless, the number of lacking references is different according to the markers: 110 species for cyt*b* and 157 species for 12S compared to species already evidenced in the area [35] and this study. This also explains why some species could be detected with only one marker and not the other. It is worthy to note that missing reference sequences can be generated for species for which tissue samples can still be collected. However, this reveals impossible for species identified before the dam construction and not caught by fishing since that time. This is a major point that needs to be anticipated in the prospect of new dam projects: biodiversity reports in pre-dam surveys should now systematically include tissue sampling for DNA analyses in addition to morphological description.

Interestingly, information provided by the morphological study is highly valuable to independently confirm the presence of species detected by the marker genes assays. It could also help in identifying the sequences of some species not yet or badly referenced in DNA databases. For example, *Xenentodon* (Beloniformes) appeared as unclassified at the species level with cyt*b* study. This was unexpected because only a single species of this genus, *Xenentodon cancila*, and of this family (Belonidae), is observed in NT2 area with 12S, fishing and according to previous survey [35]. Checking the cyt*b* reference database for this group revealed a wrong and imprecisely assigned sequence retrieved from Genbank. Removing this erroneous reference allowed to restore a correct taxonomic assignment to *Xenentodon cancila* for cyt*b* sequences. Combining results from various sources, i.e. multiple marker assays and morphological study, is an efficient way to improve and curate reference DNA databases, since Genbank is prone to errors.

#### Errors, false negatives or positives and difficulties for eDNA metabarcoding studies when lot of endemic species are unknown

Although efficient to detect species in water environment [15,16,19,22], eDNA metabarcoding studies are known to be prone to false-positive or false-negative errors [21,48,58]. In particular, cautions during the sampling and the molecular experiments need to be undertaken and various controls performed to monitor contaminants, as it was carried out for this study. To strengthen the results, the survey sampling strategy was designed to have numerous replicates in space (each river was sampled at 4 different locations, with even two samples for the reservoir), in time (samples were collected every 6 months) and in experiments (2 markers were amplified twice in duplicate for each sampling point). The level of sampling effort required is of importance to increase the detection rate of species. The choice to sample 6 litres of water by site at the starting of this survey in 2014 proved to be relevant as a similar study in the River Murray in South Australia found that at least 5 litres of water by sample were necessary to obtain a successful and complete report [30].

Recently proposed to better estimate false-positive errors rate [58], statistical analyses based on detection/non detection matrices were however not applied on the results of this study. Indeed, multiscale SODM (site-occupancy-detection modelling) still needs improvements to be used on such complex dataset with multiple samples by site, by time, and using various markers [48]. Nevertheless, false-positives inducing multiple detection events, that would arise from cross-contamination between sampling campaigns, have been limited by spreading the experiments over time. Species only detected once during the survey (19/71 for cyt*b* and 17/82 for 12S), similarly raise the question of possible false-positives. Even scarcely observed, the presence of those species make sense and are highly probable. Indeed, many of them are either detected by the other approaches or have been described in the NT2 area [35]: 12 for cyt*b* and 12 for 12S. In addition, remaining species (7 for cyt*b* and 3 for 12S), although not previously referenced in the area, have been already evidenced in Lao PDR by different sources [59–61]. Only two species (*Ptychidio jordani* (China) and *Garra rufa* (Middle-East)) evidenced by 12S could be artefacts or wrongly assigned (see above) as their distribution is inconsistent with current knowledge.

By making the choice to not take into account taxa with less than 5 reads, some information and species detection are undoubtedly missed and this increases the false-negatives rate. However, this conservative approach, in conjunction with the fact to consider only high score of BLAST identity, was taken as a compromise to identify the most relevant species in the area and to avoid “noise” in the eDNA metabarcoding data (errors, contaminants). The goal of this preliminary survey was indeed methodological so as to improve tools for future biodiversity monitoring. Another work is underway to analyse in details the evolution of fish diversity in the NT2 area at the biological level and for conservation purpose.

### Comparison between surface gillnet and eDNA metabarcoding to record species richness

Nine out of the 11 orders and 17 out of the 19 families, found by surface gillnet (Table 2) were observed by the eDNA metabarcoding approaches (Tables 3 and 4) during the entire survey. The two orders and families missed by eDNA are represented by a single species: (i) *Brachirus harmandi*, a Pleuronectiformes from Soleidae family, and (2) *Parambassis siamensis*, an Ambassidae representative for which the classification remains unclear and identified sometimes as a Chandidae [13]. However, this result is not a surprise as no 12S and cyt*b* sequences are available for both species in the reference databases. Checking the 12S sequences identified with low BLAST score, the same Ambassidae sequence is found in almost all sites of NTH or XBF (25 sampling points covering all sampling campaigns). The low BLAST identity score (around 90%) observed against the reference in the database coming from a different genus (*Chanda* sp.) explains why it was not retained in our restrictive dataset. This sequence could probably come from *Parambassis siamensis*, since it is the unique species from this group detected in the area, but this should be confirmed independently. Nevertheless, the same is not true for *Brachirus harmandi* since no Soleidae, or even Pleuronectiformes, sequences with BLAST score below the threshold fixed were observed although some Soleidae taxa are present in the 12S and cyt*b* reference databases. Finally, a supplementary order and family (Clupeiformes, Clupeidae, Tables 3 and 4) is detected with both DNA markers but is not recorded by fishing.

At the genus level, 56% of the genera are identified overall by the 3 surveys taken independently. However, nearly 90% of the genera identified using morphology criteria are found in common with eDNA when both metabarcoding assays are combined. This highlights one more time the interest of multiple marker approaches to improve biodiversity record [28,29,31]. Indeed, the results of both markers studies taken together clearly detect more species (122, not counting unclassified taxa that suggest even more diversity) than fishing (93; Fig 4). In particular, preliminary data seem to indicate that the eDNA metabarcoding surveys would allow to track benthic species not caught by surface gillnets set. Interestingly, 75% of the species captured by net and having a DNA reference (82), are evidenced by eDNA metabarcoding approach (62 species). Conversely, this means that this approach missed 25% of the fished species. This could be partly explained by an insufficient sequencing depth, and should be taken into account for future eDNA metabarcoding monitoring. Alternatively, efficiency of the primers could also be questioned for the detection of some species, such as *H*. *macrolepidota* missed with cyt*b* primers. The cyt*b* primers could be refined for future sampling campaigns but more interestingly, a third marker could be added such as 16S rDNA [30] or another 12S fragment adjacent to the one we used [25]. This last set of universal primers (MiFish), published after the start of this project, seems to improve fish taxonomic resolution [25]. Nearly twice longer than the 12S fragment we targeted in this study, the robustness of the amplification may however be reduced [19].

Considering the pre-dam surveys of Kottelat [35], eDNA metabarcoding approach detects 81 species previously described in NT2 area, among which 30 were not evidenced by surface gillnets at the same time period (Fig 4A). This includes for example the Clupeidae family mentioned above. Thus, 60% of Kottelat species having a DNA reference (81/135; Fig 4B) are retrieved by 12S and cyt*b* assays combined. By comparison, monitoring by surface gillnet only provides 34% of the previously morphologically described species (74/219; Fig 4A) and less than 50% of the ones with DNA references (67/135; Fig 4B). eDNA metabarcoding being more sensitive [16,19], it could indicate that population size has been reduced for some species. However, this result should be confirmed by independent studies.

Interestingly, this survey highlights the presence of species not reported before the dam construction, among which 11 species detected by both monitoring methods. Eight are specifically observed in the XBF and 2 others, potentially invasive exotic species, the tilapia (*Oreochromis niloticus*) and the common carp (*Cyprinus carpio*), are present in the two rivers. Of note, 30 more species are specifically detected by eDNA metabarcoding in the NT2 area, revealing 4 additional exotic species known to have been introduced into Lao PDR through various sources [61]. The 14 species spotted by cyt*b* are coherent with a presence in Lao PDR. However, 10 species evidenced by 12S only are geographically dubious and probably correspond to close species and not real ones as already discussed above in the ‘limits’ paragraph for the 12S marker.

### The XBF and NTH have different profiles and species richness

Both rivers are very different and originate in various locations along the Laos-Vietnam boundaries. On one hand, NTH and its tributaries have a high gradient with numerous large rapids and waterfalls before reaching the Nakai Plateau [35]. There, the river joins the reservoir impounded in 2008. Downstream of the dam, a minimum environmental flow is maintained to supply the NTH [6]. On the other hand, the XBF has a typical morphology of highland river without visible strong gradient or large rapids [35]. This large river is relatively shallow with slow flowing and was previously characterized by large quantities of sand and suspended solids during wet season in the middle and lower part of the river [35]. However, the river hydrology has changed since the commercial operation. Indeed, the XBF received now the turbinated waters coming from the reservoir by the DSC (Fig 1). This leads to modification in the water characteristics in the XBF after the confluence with the DSC [6] occurring between XBF1 and XBF2 sites. This change is particularly significant during the dry season.

The surveys performed by Kottelat to evaluate the fish biodiversity in the NT2 area before the dam construction pointed out main differences between both rivers ([35] for review). Although less species were recorded (74 species), NTH showed a large proportion of endemic species (28%). On the other hand, the species observed in XBF (178 species) were very diverse and typical of other main tributaries of the Mekong in Laos and only 5% were endemic. This mainly explains why 71% of the species specifically observed in NTH have no DNA references (29/41) whereas this percentage is only 35% for XBF.

The monitoring by surface gillnets (Table 2) and by eDNA metabarcoding (Fig 2) performed for this study (2014–2016), confirm striking differences between NTH and XBF and reach highly similar conclusions. Cypriniformes is the largest group in terms of species (>62 in total) but also in abundance (>70%, Table 2) and is observed in both rivers. The Siluriformes is the second group of species represented (>15 in total) with more species evidenced in the XBF when compared to the NTH. Some representatives of Tetraodontiformes, Osteoglossiformes and Beloniformes are specifically found in XBF, while Gobiiformes is exclusively observed in NTH. Among the 93 species captured during the study, 14 were detected solely in NTH and 62 in XBF (8 and 50 for cyt*b*; 13 and 45 for 12S respectively). In particular for NTH, the reservoir appears with the poorest taxonomy variety (Fig 2) with only few species present at high frequency (notably *H*. *macrolepidata* (12S) and *Oreochromis niloticus* (cyt*b*), similarly observed as highly abundant by fishing [12] at this sampling site). Despite missing data in DNA reference databases, more than half the species were confirmed on their specific location in a single river by at least one marker assay. Details about the ecological implications of these differences are beyond the scope of this methodological paper and will be discussed elsewhere.

## Conclusions

This study allows describing a complete methodological procedure to perform a most comprehensive biodiversity report with multidisciplinary approaches in a hydropower reservoir context with high endemism. Tested along time and validated from the sampling step to the analyses of the data, this procedure has been optimized to reduce experimental time and cost. It should be useful to anticipate the fish report and monitoring needed for the future dam projects that would concern similar ecosystems in the Mekong basin. In particular, the survey highlights the necessity to include DNA sampling of newly described or threatened species when performing pre-dam biodiversity report. The use of eDNA metabarcoding studies allowing a non-invasive, sensitive and efficient biodiversity monitoring with a low cost will most likely be integrated routinely into biomonitoring programs over the next decade [19,31,62].

## Supporting information

S1 File Supplementary information. (PDF)

S1 Table Names of the sampling locations and geographic coordinates (modified from (33)). (PDF)

S2 Table Detailed listing of taxa detected by surface gillnet capture survey, 12S study (%BLAST identity >97%) and cyt*b* analysis (%BLAST identity >95%) with only classified species. The number of campaigns (C1 to C5) where the species have been detected is given. Their detection in NTH or/and XBF is indicated by 1, their absence by 0. The sampling sites are composed of 4 NTH sites and 4 XBF sites.
(XLSX)

S3 Table Taxonomic repartition of 12S and cyt*b* reads obtained for all filters (all sites and all campaigns). Only reads were %BLAST ID was higher than 97% for 12S and 95% for cyt*b* were considered. The number of groups observed at different taxonomic levels is given when the determination was clear. The numbers in parentheses give the potential supplementary groups for which no clear taxonomic assignment was available. Only species for which at least 5 reads were obtained are considered. The number of reads and their percentage are also given.
(PDF)
