# Supplementary material for: Direct fishing and eDNA metabarcoding for biomonitoring during a 3-year survey significantly improves number of fish detected around a South East Asian reservoir
Source: PLoS One. 2018 Dec 13;13(12):e0208592. doi: 10.1371/journal.pone.0208592 (PMC6292600; doi:10.1371/journal.pone.0208592)
Supplement: S1 File — (PDF) [file pone.0208592.s001.pdf]

Supplementary Information

Detailed protocols and tests performed for the eDNA metabarcoding study

Cautions to perform the eDNA study in Lyon

To prevent samples from DNA contamination, DNA extraction, PCR set-up and post-PCR analysis were conducted in three different dedicated and physically isolated laboratories. The technical procedures were those generally used for ancient/degraded DNA studies: the personnel involved wore lab suits and sterile latex gloves and used a strict go-forward process to work from the less DNA contaminated room to the most amplified DNA contaminated one without coming back during the same experiment. Moreover, DNA extraction and PCR set-up labs were UV irradiated at 254nm, the day before use and after the experiments, and cleaned with DNA-ExitusPlus (Pan Reac AppliChem).

DNA extraction from filters

As an initial step, the frozen filters were crushed using a scalpel and reduced into small pieces constituting the material to be used for the DNA extraction. Several tests were performed in order to identify the most reliable way to extract the DNA from filters.
To start, the Nucleospin Soil kit (Macherey Nagel) was used and tested with different combination of buffers following the manufacturer's recommendations. These preliminary tests showed that DNA extractions conducted using Lysis Buffer SL1 and Enhancer SX led to a better efficiency for subsequent and downstream PCR amplifications. This protocol was thus retained for all the filters extractions.
Then, two main DNA extraction protocols were tested on the C1 and C2 samples. They differed by the way to treat the filters before the DNA extraction. In the first approach, 100 to 300mg of fragmented filters were homogenized in a tube containing ceramic beads in 700µl of SL1 Buffer and 150µl of enhancer SX by using the Precellys®24 homogenizer (two cycles, 6500rpm – 15s). The DNA extraction and purification were then performed with the Nucleospin Soil kit according the manufacturer's recommendations. In the second approach, all the remaining crushed filters were incubated overnight at 37°C with 5ml of SL1 buffer and under gentle agitation. After centrifugation (5min, 11000rpm) to pellet the debris, 1400µl of supernatant and 300µl of enhancer SX were mixed in a tube containing ceramic beads and homogenized using the Precellys®24 (two cycles, 6500rpm – 15s). The volume of SL3 buffer was adjusted to 300µl for the precipitation of contaminants, and two successive loading of the supernatant (2x 700µl) were carried out onto the Nucleospin inhibitor removal column. After combination of flow-throughs, a volume of 500µl of buffer SB was added to adjust the binding conditions. Three successive loading of the supernatant (3x 550µl) were done onto the Nucleospin Soil column. The next steps were performed according to the manufacturer's recommendations in the same manner than the first approach.

As the second approach gave significantly better results for the C1 and C2 samples by detecting much more taxonomic diversity in all samples, filter samples from sampling campaigns referenced C3, C4 and C5 were extracted with the second approach only.
To monitor possible DNA contamination of reagents, a mock extraction was performed in each extraction session (distributed in time according to sampling campaigns) constituting an extraction blank control.

Metabarcoding, PCR amplification and sequencing

Barcoded fusion primers (i.e. containing the sequences of Ion Torrent A and P1 adapters) were used to allow the construction of libraries during the PCR amplification step. Short mitochondrial fragments of the 12S RNA (hereafter 12S) and cytochrome b (cyt*b*), around 100 bp and 235 bp in length respectively, were targeted. Because the primer pairs can amplify either all vertebrates (12S; [1]) or vertebrates but more preferentially fish (cytb; [2], with a single base modification and [3]), blocking primers were used to prevent human DNA amplification.
All DNA amplifications were carried out in a final volume of 25µl, using Taq environmental Master Mix 2.0 (Thermofisher), 0.4µM of each primer, 4µM of human blocking primer and 2µl of DNA extract (template) or ultra-pure water for negative controls. The PCR program was as follow: 10min at 94°C, 50 cycles of 30s at 95°C, 1 min at 55°C (12S) or at 50°C (cyt*b*) and 30s at 72°C, and a final elongation during 7min at 72°C. To monitor DNA contamination of PCR reagents or by aerosols, PCR blank controls were included in each PCR session. Two independent PCR sessions were performed by sample, each including at least two replicates. The same barcode was used for a sample for both markers and replicates. After gel electrophoresis analysis, positive PCR products were purified using the Nucleospin PCR Clean-up kit (Macherey Nagel) and their concentration estimated with a Qubit 2.0 Fluorometer using the HS DNA kit from Thermofisher. For each marker and sample, amplicons were pooled all together (75ng per sample). As we used fusion primers, dimers exceed the size of fragments usually eliminate by the cleaning columns (<50bp). To eliminate those dimers that can hamper the sequencing results by generating many small uninformative reads, DNA libraries were separated by electrophoresis and excised from agarose gel using a scalpel. A first purification was done with a Nucleospin Gel Clean-up kit (Macherey Nagel) followed by a second one using Ampure beads (Beckman Coulter; ratio of Beads/DNA in solution: 0.9x). Quantitation and quality assessment of libraries were performed on 2200 Tapestation analyzer using the High Sensitivity D1000 ScreenTape kit (Agilent Technologies). Each barcoded library (12S and cyt*b*) was diluted to 100pM and at most 12 samples mix all together in an equimolar manner. Template preparation procedure and sequencing followed the Ion PGM standard protocols from Thermofisher (Ion PGM^{TM} Hi-Q^{TM} OT2 and Ion PGM^{TM} Hi-Q^{TM} Sequencing Kits). Eight runs on 318v2 chips were done for this project and sequenced on a Ion Torrent PGM.
The raw data (fastq files) for each of the 40 conditions analysed in this study (8 sites sampled during 5 sampling campaigns) and for each marker (12S and cyt*b*) have been deposited to SRA under accession PRJNA496021.

Adding new sequences of endemic species

As many species found in the Nam Theun area are endemic species, we decided to enrich the database by sequencing the 12S and cyt*b* for some of them. We used the flesh samples collected during the C1 and C2 sampling campaigns (see *Fish monitoring*) corresponding to 66 species and 228 individuals. Species for which sequences were not available in Genbank for at least one marker, or seemed dubious, were identified. Two individuals by species were considered when possible.

DNA was extracted using the Nucleospin Tissue kit following the manufacturer's instruction (Macherey Nagel). Using 19 available fish sequences of the Nam Theun area, a first primers set was designed to amplify around 9.6kb of the mitochondrial genome: COMP_MITO_FOR2_9550 (5' TGATGAGGMTCATAATCTTTCTAGTAT 3') and COMP_MITO_REV2_2590 (5' GAACTCAGATCACGTAGGACTT 3'). Long Range PCR was performed with 2 to 5µl of DNA extracts using the LongAmp Hot Start Taq 2X Master Mix kit (New England Biolabs). PCR reactions were done in 25 µl following the program: 94°C for 30s, 40 cycles of 30s at 94°C, 1min at 55°C and 10min at 65°C, and a final elongation during 10min at 65°C. This first PCR was diluted 10 times and re-amplified with two different primers pairs targeting 750bp of 12S and 910bp cyt*b* (including primers). The primers were designed as before, taking advantage of available sequences, and fused with M13-26REV (CAGGAAACAGCTATGAC) or T7-REV (TAGTTATTGCTCAGCGGTGG) for easiest Sanger sequencing. The fused primers pair were: M13-26REV_Seq12SF1 (5' CAGGAAACAGCTATGACCGGTAAAACTCGTGCCAG 3') used with T7-REV_Seq12S_R1 (5' TAGTTATTGCTCAGCGGTGGCACCTTCCGGTACACTTAC 3') and M13-26REV_SeqCytb_F1 (5' CAGGAAACAGCTATGACAAAATYGCWAACGACGCACT 3') used with T7-REV_SeqCytb_R1 (5' TAGTTATTGCTCAGCGGTGGCCTCGTTGTTTDGAGGTGTG 3'). 2µl of 1/10 diluted first-PCR was mixed with Taq Environmental Master Mix 2.0 (Thermofischer) and 0.4µM of each primer for a final volume of 25µl and amplified with the following protocol: 94°C for 10min, 35 cycles of 30s at 94°C, 30s at 55°C (12S) or 30s at 50°C (cyt*b*) and 1min at 72°C, and a final elongation during 10min at 72°C. When necessary, products were purified to remove artefactual small band (cyt*b*) with Ampure beads (Beckman Coulter; ratio of Beads/DNA in solution: 0.6x). Products were sent to Genewiz (https://www.genewiz.com) for Sanger sequencing in both strand using the M13-26REV and T7-REV universal primers.

The sequences obtained were added to the 12S (62 sequences) and cyt*b* (59 sequences) reference databases. These sequences are available in Genbank under the accession numbers MH688181 to MH688301.

Contaminants detected in controls:

Experiments were performed in dedicated rooms, where amplified DNA is not present, and by performing various controls all along the process, from the sampling to the sequencing. All positive amplicons obtained from these controls were sequenced with the samples and revealed

no or few contaminants in extraction and PCR controls of different kinds (negative, aerosol) excepted some reads of human (for cyt*b*) or pig (for 12S). In the case of pure-water used to rinse the tanks, sporadic contaminants were detected, usually of a single species that was different for each sampling campaign (human in C2 (cyt*b*) or C4 (cyt*b* or 12S), pig in C3 (12S) or a single fish species (for 12S : *Scaphiodonichthys acanthopterus* in C1, *Oxyeleotris marmorata* in C2, *Hypsibarbus vernayi* in C3 ; for cyt*b* : *Oreochromis* in C1). Even if detected in the pure-water, reads of those species were considered for the samples analyses because: i) those species were not observed in the other controls performed meaning that no contamination occurred at the extraction or PCR steps ; ii) those species were not systematically detected in all the samples from the involved sampling campaign, and sometimes even not detected at all, meaning that the contaminant was only transient and most probably did not affect the sampling. Only *Hypsibarbus vernayi* was present in the pure-water and in all C3 samples for 12S but with a variable coverage and a number of reads below 20 for 3 of them (XBF0, XBF1, XBF3). Because those numbers were slightly higher that the threshold of 5 reads that we fixed to consider the presence of a species with confidence, the reads of this species in the filter samples of the C3 sampling campaign were kept for further analyses but with this warning in mind.

Threshold used to consider species assignation:

The pairwise differences computed between sequences of the reference databases coming from individuals of the same species or from individuals of the same genus were analysed. Their distributions were observed for both markers (not shown). By taking into account the length of the fragment amplified and the intra-species and intra-genus diversity observed, threshold of 97% and 95% of blast similarity for 12S and cyt*b* respectively were considered for the assignment of reads at a species level with better confidence. In other words, only reads for which a match was obtained in the reference databases with a blast identity score higher or equal to 97% and 95% for 12S and cyt*b* respectively were considered at first. After cleaning and application of the threshold, around 8.5M reads were kept for analyses with about 5 times more 12S sequences than cyt*b*.

References:

1. Riaz T, Shehzad W, Viari A, Pompanon F, Taberlet P, Coissac E. ecoPrimers: inference of new DNA barcode markers from whole genome sequence analysis. Nucleic Acids Res. 2011;39(21):e145.

2. Miya M, Nishida M. Use of Mitogenomic Information in Teleostean Molecular Phylogenetics: A Tree-Based Exploration under the Maximum-Parsimony Optimality Criterion. Mol Phylogenet Evol. 2000;17(3):437-55.

3. Minamoto T, Yamanaka H, Takahara T, Honjo MN, Kawabata Z. Surveillance of fish species composition using environmental DNA. Limnology. 2012;13(2):193-7.
