# Supplementary material for: Direct fishing and eDNA metabarcoding for biomonitoring during a 3-year survey significantly improves number of fish detected around a South East Asian reservoir
Source: PLoS One. 2018 Dec 13;13(12):e0208592. doi: 10.1371/journal.pone.0208592 (PMC6292600; doi:10.1371/journal.pone.0208592)
Supplement: S1 Table — (PDF) [file pone.0208592.s002.pdf]

S1 Table: Names of the sampling locations and geographic coordinates (modified from [33])

Water body	Sampling sites	Description location	Possible effect of the dam/reservoir	Latitude	Longitude
Nam Theun River	NTH1	Nam Theun river upstream Nam Noy confluence	Reference station, not impacted by the reservoir. Upstream of the reservoir backwaters	17.8194359	105.3470586
	NTH2	Nam Theun river upstream reservoir, downstream Nam Theun / Nam Noy confluence	Reference station, almost not impacted by the reservoir. Seasonally under the influence of the Reservoir.	17.7381281	105.3228492
	NTH6	Nam Theun 10 km upstream Nam Theun bridge	Impacted, located after the dam	18.1859092	104.7157990
Nam Theun 2 Reservoir	RES4	Reservoir at Ban Thalang	Middle of the reservoir. Narrow waterway. Transition between east and west part of reservoir	17.8509318	105.0457535
Xe Bangfai River	XBF0	Upstream confluence of the Downstream Channel	Reference stations upstream of the downstream channel et outside of the backwaters.	17.4688875	105.5074783
	XBF1			17.5000469	105.4257345
	XBF2	Mahaxai, downstream confluence with the Downstream Channel	Receiving the turbinated waters from the Downstream Channel.	17.4250942	105.2030290
	XBF3			17.3377207	105.1522599
