# Supplementary material for: Direct fishing and eDNA metabarcoding for biomonitoring during a 3-year survey significantly improves number of fish detected around a South East Asian reservoir
Source: PLoS One. 2018 Dec 13;13(12):e0208592. doi: 10.1371/journal.pone.0208592 (PMC6292600; doi:10.1371/journal.pone.0208592)
Supplement: S3 Table — Only reads were %BLAST ID was higher than 97% for 12S and 95% for cytb were considered. The number of groups observed at different taxonomic levels is given when the determination was clear. The numbers in parentheses give the potential supplementary groups for which no clear taxonomic assignment was available. Only species for which at least 5 reads were obtained are considered. The number of reads and their percentage are also given. (PDF) [file pone.0208592.s004.pdf]

S3 Table. Taxonomic repartition of 12S and cyt*b* reads obtained for all filters (all sites and all sampling campaigns).

	Taxonomic group	Order	Family	Genus	Species	Reads	%reads
**12S (97%ID)**	*Fish*	9 (+1)	22 (+4)	68 (+12)	82 (+34)	6654996	95.68
	Amphibians	1	6	7	2 (+5)	56139	0.81
	Mammals	4 (+1)	13 (+1)	20 (+4)	15 (+12)	221119	3.18
	Birds	4	5	6 (+2)	6 (+2)	23560	0.34
	Reptiles	-	-	-	-	-	-
**Cytb (95%ID)**	*Fish*	7 (+1)	15 (+1)	58 (+7)	70 (+32)	1149768	94.35
	Amphibians	1	2	2	2	1703	0.14
	Mammals	3 (+1)	4	5 (+1)	4 (+4)	65361	5.36
	Birds	2	3	3	3	864	0.07
	Reptiles	1	2	2	2	951	0.08
**12S and Cytb combined**	*Fish*	10	25 (+2)	91 (+14)	124 (+53)	7804764	95.48
	Amphibians	1	6	9	4 (+5)	57842	0.71
	Mammals	4 (+1)	14 (+1)	21 (+5)	17 (+14)	286480	3.50
	Birds	4	7	8 (+2)	8 (+2)	24424	0.30
	Reptiles	1	2	2	2	951	0.01

Only reads were %Blast ID was higher than 97% for 12S and 95% for cyt*b* were considered. The number of groups observed at different taxonomic levels is given when the determination was clear. The numbers in parentheses give the potential supplementary groups for which no clear assignation was available. Only species for which at least 5 reads were obtained are considered. The number of reads and their percentage are also given.
